# IFITs: Emerging Roles as Key Anti-Viral Proteins

**DOI:** 10.3389/fimmu.2014.00094

**Published:** 2014-03-10

**Authors:** Gregory I. Vladimer, Maria W. Górna, Giulio Superti-Furga

**Affiliations:** ^1^Laboratory of Giulio Superti-Furga, Center for Molecular Medicine of the Austrian Academy of Sciences, Vienna, Austria

**Keywords:** IFIT, innate immune system, anti-viral immune response, TPR, PAMPs

## Abstract

Interferon-induced proteins with tetratricopeptide repeats (IFITs) are a family of proteins, which are strongly induced downstream of type I interferon signaling. The molecular mechanism of IFIT anti-viral activity has been studied in some detail, including the recently discovered direct binding of viral nucleic acid, the binding to viral and host proteins, and the possible involvement in anti-viral immune signal propagation. The unique structures of some members of the IFIT family have been solved to reveal an internal pocket for non-sequence-specific, but conformation- and modification-specific, nucleic acid binding. This review will focus on recent discoveries, which link IFITs to the anti-viral response, intrinsic to the innate immune system.

## Introduction

The germline-encoded innate immune system initiates a fast and targeted response upon recognition of an invading virus. Most research on the innate immune system is focused on how the host senses and detects pathogen-associated molecular patterns (PAMPs), including viral nucleic acids. Foreign nucleic acid within the cytosol is an extremely potent PAMP, and the detection elicits a strong innate immune response (1). The host proteins that sense viral PAMPs, termed pattern recognition receptors (PRRs), range in their specific targets enormously and are located in endosomes and the cell cytosol (2–4). Some important PRRs that are specific to virus nucleic acid recognition include Toll-Like Receptors (TLRs) TLR3, TLR7, TLR8, and TLR9 (4, 5). Absent in Melanoma 2 (AIM2)-like receptors such as the AIM2 inflammasome and IFI16 (6–9), and RIG-I-like receptors including MDA5 (melanoma differentiation associated gene 5) and RIG-I (retinoic acid inducible gene I) (10, 11). The detection of various viral nucleic acid species by these receptors elicits signaling cascades that include the production of anti-viral genes and pro-inflammatory cytokines, including type I interferons (IFNs) (1). These responses slow virus replication by setting in motion a systematic anti-viral response.

Type I IFNs are comprised of IFNα and IFNβ; these are responsible for an array of biological and immunological functions [reviewed in Ref. (12)]. Type I IFN signaling is mediated via the IFNα/β receptor (IFNAR), and downstream signaling results in the upregulation of IFN-stimulated genes (ISGs) (12). Beginning with the innate immune sensing of virus infection, ISGs encode many important protective and anti-viral pathways. ISGs are directly responsible for blocking virus infection and priming pro-inflammatory and adaptive immune response systems (12).

While the ISG class is quite large and diverse, this review will focus on a family of proteins that recently emerged as having a wide range of anti-viral functions: interferon-induced proteins with tetratricopeptide repeats (IFITs). The detection of virus infection by receptors and downstream pathways is of fundamental importance for our understanding of innate immunity processes regulating cellular homeostasis. Moreover, innate mechanism leading to inhibition of virus replication is particularly worth being investigated as they can possibly be harnessed for anti-viral therapy design.

## The IFIT Protein Family

The IFIT family includes four canonical human members (IFIT1, IFIT2, IFIT3, and IFIT5) and three mouse members (IFIT1, IFIT2, and IFIT3), which are induced upon simulation with IFN, virus infection, or other PAMP recognition (13, 14). Another human IFIT, IFIT1B, is thought to be expressed in a non-IFN dependent manner due to lack of an interferon-stimulated response element (ISRE), which typically are present in two to three copies within the promoters of the other IFIT genes (15, 16). IFIT5 is a paralog of IFIT1, which is absent in the murine genome. Instead, another closely related gene seems to be present in the mouse, Ifit1c (17). Moreover, two additional genes, Ifit1b and Ifit3b, are part of the mouse repertoire. IFIT homologs have been discovered in many vertebrate species: birds, fishes, and amphibians (15, 18). Their conserved role throughout evolution hints to their general importance. Since fish commonly contain multiple copies of IFIT genes that most resemble IFIT1/5, it is tempting to speculate that these IFITs are part of an ancient immune defense mechanism in vertebrates (19).

While IFITs generally are not expressed in cells at high basal levels, the transcription of IFIT genes rapidly increases during virus infection or IFNAR signaling (16). Low levels of IFIT5 expression, however, have been detected in HEK cells, which further increase several-fold upon IFN stimulation (13). The presence of the ISREs within the IFIT promoter region explains their low baseline-transcriptional levels and fast IFN-dependent induction (16). However, the kinetics of the transcriptional levels of specific IFITs can be cell line and tissue dependent (20, 21). Moreover, the transcriptional profile in different cell types could imply that specific IFITs have various functions during virus infections in the host.

## IFIT Structure

All IFIT proteins consist of repeats of the eponymous tetratricopeptide (TPR) motif, which typically contains 34 amino acids with the consensus sequence [WLF]-X(2)-[LIM]-[GAS]-X (2)-[YLF]-X(8)-[ASE]-X(3)-[FYL]-X(2)-[ASL]-X(4)-[PKE] that adopts a basic helix-turn-helix fold. Adjacent TPR motifs usually form a sheet of antiparallel helices that curves into a super-helix, and this unique fold presents concave and convex curved surfaces that allow for binding of diverse ligands. TPR domains are conserved in all kingdoms of life and are generally believed to serve as protein and peptide recognition domains; with the discovery of RNA-binding IFITs, the known ligand spectrum of TPR motifs is broadened to also include nucleic acids.

**Figure 1:**
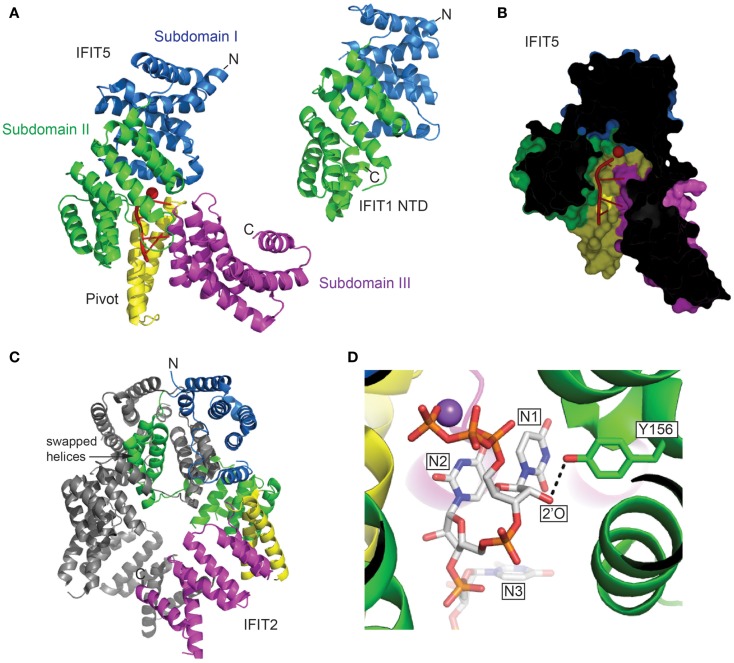
**(A)** The structures of human IFIT5 in complex with oligoA and human IFIT1 NTD in cartoon representation (PDB entries 4HOT and 4HOU). The subdomains identified in IFIT5 are color coded, and RNA is in red. **(B)** Cross-section of the complex of IFIT5 with oligoA, showing a narrow pocket that binds four nucleotides. **(C)** The structure of human IFIT2 (PDB entry 4G1T) colored according to the corresponding IFIT5 subdomains. Indicated are helices 7–9 that are swapped with the other protomer. **(D)** The Y156 of IFIT5 forms a hydrogen bond (dotted line) with the 2′-*O* of the first ribose (IFIT5 in complex with oligoU, PDB entry 4HOS). Metal ions are depicted as spheres (Red, Mg^2+^; Purple, Na^2+^).

The recent crystallographic structures of IFIT5 and the N-terminal half of IFIT1 (NTD) (22–24) reveal that the usual TPR super-helix is interrupted by an upside-down flip of the N-terminal subdomain, but nevertheless the remainder of the protein forms a concave surface that binds the 5′ end of RNA (Figure 1A). The narrow pocket can accommodate up to four nucleotides of exclusively single-stranded RNA (Figure 1B), and the C-terminal subdomain tightens slightly around the aperture upon ligand binding. Most notably is the pocket that engages the 5′ triphosphate extension of the single-stranded RNA. Whereas, IFIT5 is monomeric, the structure of IFIT2 reveals a dimer (25), and IFIT1, IFIT2, and IFIT3 form homodimers in solution (13). The dimerization of IFIT2 occurs through swapping of three helices belonging to TPR motifs 3 and 4 (Figure 1C). IFIT2 might have a preference for double-stranded RNA (25), but it is not clear where the RNA-binding interface is located. With IFIT1 and IFIT5 targeting the 5′ end, and IFIT2 binding the body of the RNA, the IFIT proteins have diversified in the features of the non-self RNA that they recognize, described below, but it remains to be shown whether they complement each other and act in concert in a synergistic manner.

## IFIT Anti-Viral Function

Since IFITs are swiftly induced following virus infection, it is hypothesized that IFITs play a role in the anti-viral milieu of cells. Over the past years, many investigations have alluded to the important anti-viral mechanisms of each IFIT family member. Below, we will discuss the most important findings.

### 5′-Triphosphorylated and 2′-*O*-unmethylated capped RNA binding

**Figure 2:**
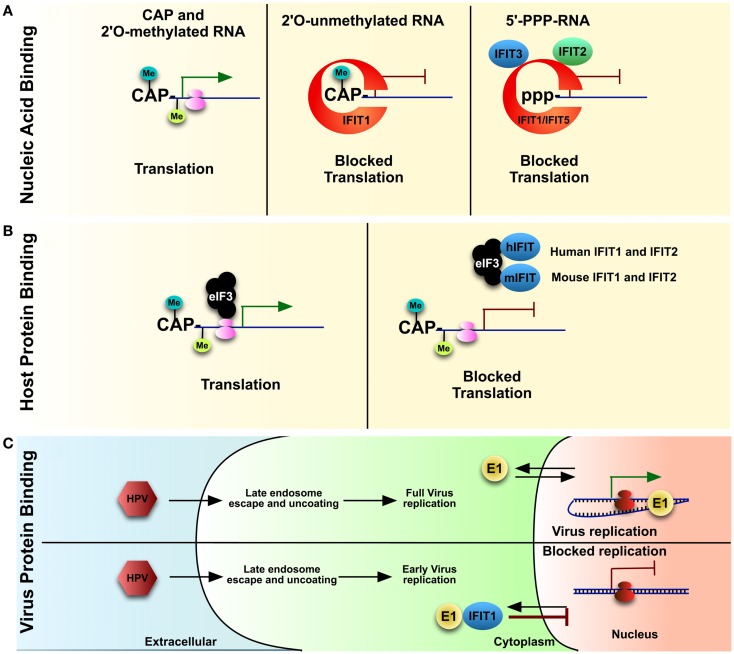
**IFITs, once upregulated due to IFN signaling, play various roles in blocking virus and host protein translation**. IFIT1 can bind to the **(A)** 5′ of mis-modified RNA either which is 2′-*O*-unmethylated or has 5′PPP-RNA (features of foreign nucleic acid) vs. properly capped host mRNA. IFIT1, along with a complex of IFIT2 and IFIT3, can block translation of these nucleic acids. Multiple IFITs have been described to bind to various subunits of host **(B)** eIF3, a key component of mRNA translation. Though the target of eIF3 is properly processed mRNA, including host mRNA, IFIT1 may block translation cell-wide during virus infection. As well, IFIT1 can bind **(C)** a key virulence factor of HPV, helicase E1, and sequester it into the cytoplasm, thereby preventing virus replication.

In general, cellular cytoplasmic RNAs are single stranded and contain a 5′-monophosphate or *N*-7-methylated guanosine cap linked by a 5′-to-5′ triphosphate bridge to the first base: rRNAs/tRNAs and mRNAs, respectively. In higher eukaryotes, mRNA is further modified with a methylated 2′-*O* position of the first ribose (26, 27). These modifications and secondary additions assist in not only translational control, but the lack thereof plays a role in the detection of foreign nucleic acid. In contrast, viruses may form long double-stranded RNA, and/or generate triphosphorylated RNA (PPP-RNA) during their life cycle, which elicits a strong anti-viral response (28, 29). Using a proteomics approach, with PPP-RNA as bait, a mass spectrometry analysis revealed IFIT1 as a major binding partner in HEK cells (13); thereby revealing a role for IFIT1 in recognizing and potentially sequestering viral PPP-RNA, preventing it from being translated by the host machinery (Figure 2A). From the proteomic and subsequent biochemical analysis, it appeared as if only IFIT1 would bind the PPP-RNA directly while IFIT2 and IFIT3 bind IFIT1 in a multi-protein complex required for anti-viral activity (13). Knocking down IFIT1, IFIT2, and IFIT3 in HeLa cells with siRNA resulted in an increase rate of infection by viruses known to display a PPP-RNA nucleic acid species during their life cycle such as Rift Valley fever virus (RVFV), vesicular stomatitis virus (VSV), and influenza A. In contrast, growth of Encephalomyocarditis virus (EMCV), which does not produce a PPP-RNA species, was unaffected by the presence or absence of IFIT1 (13). Moreover, studies with IFIT1^−/−^ mouse fibroblasts and myeloid cells also resulted in increased replication of VSV, with no changes detected in pro-inflammatory cytokines (13). Also, other studies determined that IFIT2 protects mice from VSV neuropathogenesis (30), and IFIT3 expression in human A549 cells is required for IFNα-dependent anti-viral activity against VSV (31).

As described above, higher eukaryotes and many viral RNAs are not only methylated at the *N*-7 position, but also the 2′-*O* of the 5′ guanosine cap. The lack of the latter cap, common for foreign nucleic acids, elicits a strong anti-viral response (32). Viruses lacking this 2′-O-methylation, such as a West Nile virus (WNV) that lacks 2′-*O*-methyltransferase activity, were unable to infect wild type cells, but could replicate in cells lacking IFIT1 expression (33, 34). Again, using proteomics, IFIT1 was discovered to have a much stronger affinity for 2′-*O* uncapped vs. capped RNA, which explains the IFIT1 mediated control of 2′-*O*-methyltransferase deficient WNV (17). The study found that IFIT1 bound 2′-*O*-unmethlyated RNA, which resulted in an inhibition of translation, and therefore decreased virus infection (Figure 2A). Human IFIT5 has also been described to bind PPP-RNA (outside of the IFIT1–IFIT2–IFIT3 complex), and also to uncapped 2′-*O*-unmethylated RNA (13, 17) (Figure 2A). IFIT1 binding of 2′-*O*-unmethylated RNA was also supported by another study that described a role for IFIT1 in controlling Japanese Encephalitis Virus (JEV) 2′-MTase mutant by binding preferentially to capped 2′-*O*-unmethylated viral mRNAs (35).

IFIT1 can possibly accommodate capped RNA due to the larger size of the binding cavity. Mutagenesis of IFIT1 in the residue, which in IFIT5 makes contact with the 2′-OH of the first nucleotide (Figure 1D), followed by pulldowns on RNA, suggests that IFIT1 should be highly sensitive to the methylation status of this moiety (22), with methylated RNA being a poor ligand. Additionally, IFIT5 seems less sensitive to the disruption of 2′-*O* binding, which enforces the notion of its high specificity for 5′PPP-RNA, since 2′-O-methylation usually occurs in conjunction with capping; there is also little evidence of sequence specificity in binding, as demonstrated by the structures of IFIT5 in complex with oligoU, oligoC, and oligoA (22). Furthermore, IFIT family members have been shown to effect translation by binding to mRNA with various 5′-modifications (36).

These studies nicely define a role for IFITs in the preferential binding to mis- or un-modified RNA in the cytoplasm; using a key evolutionarily conserved feature of transcriptional regulation to decipher self- vs. non-self nucleic acid.

### Inhibition of viral protein translation

Eukaryotic cap-dependent protein translation relies on an *N*-7-methylguanoside cap at the 5′ end of mRNA, compared to the 5′-PPP-modified viral RNA species described above (37). Evidence has shown that IFIT family members can lessen host cap-dependent protein translation by binding to subunits of the eukaryotic initiation factor 3 (eIF3) translation complex (38) (Figure 2B). The eIF3 protein complex is required for translation initiation in several ways, including: mRNA recruitment, scanning mRNA for the start codon, and tRNA delivery to the translation machinery (37). Human IFIT1 and IFIT2 may perhaps block function of eIF3 tRNA delivery while human IFIT2 and mouse IFIT1 and IFIT2 may block mRNA recruitment (38–40). The decrease in mRNA translation can have detrimental effects on the host, though this also yields host-dependent virus replication. Viruses can also use internal ribosome entry sites (IRESs) during their replication for cap-independent translation, which also requires eIF3 (37). It was discovered that human IFIT1 can suppress this IRES-dependent viral RNA translation during Hepatitis C virus (HCV) infection (41). These studies, for which the molecular mechanism remains to be conformed, would suggest that IFIT family members may, on top of the indirect effects through RNA engagement, also affect virus translation directly, by altering translational processes itself.

### Direct viral protein binding

While it has become clear that IFIT mechanism of anti-viral activity is directed through binding foreign nucleic acid, yeast-two-hybrid studies have suggested that IFITs can bind other viral proteins. IFIT1 binds to E1, a viral helicase from Human papillomavirus (HPV), which is required for replication (42, 43). IFIT1 binds E1 and sequesters it within the cytoplasm, preventing it from aiding in viral replication within nucleus (Figure 2C). This was supported using a HPV virus expressing an E1 helicase with a deleted F399amino acid residue, which was required for IFIT1 binding; the resulting virus had no loss of replication (43).

## IFIT Role in Anti-Viral Signal Pathway Transduction

As well as being described as effector proteins in anti-viral replication, IFITs may also control downstream signaling, though some controversies exist. Pichlmair et al., who originally described the role of IFIT1 in binding PPP-RNA, noticed no decrease in type 1 IFNs produced in mouse fibroblasts, macrophages, or dendritic cells lacking IFIT1 (13). Later, IFIT1 was proposed as one of many innate immune “bottlenecks” and that the knocking down of IFIT1 resulted in decreased pro-inflammatory responses after LPS treatment of cells (44). In determining IFIT-mediated immune pathways, a role for IFIT3 to interact with TBK1 (TNFR-associated factor family member-associated NF-κB activator-binding kinase 1), an important innate immune modulating kinase (45), was outlined. This interaction of IFIT3 with TBK1 bridges the kinase with mitochondrial anti-viral signaling (MAVS) on mitochondria; over-expression or knock down of IFIT3 resulted in the increase or decrease of anti-viral gene expression, respectively.

In contrast, groups have also reported immune suppressive function of IFITs. By over-expressing IFIT2 in mouse macrophages, Berchtold et al. observed reduced LPS-induced expression of multiple pro-inflammatory cytokines including TNF and IL-6 (46). This was associated with reduced mRNA stability of the cytokine transcripts in the presence of increased IFIT2, suggesting post-transcriptional regulation of inflammatory responses (46). This phenomenon, however, could be due to the natural function of IFIT family members to bind RNA and therefore an over-expression could cause intrinsic cellular issues, as well as cell growth defects (47). Furthermore, IFIT1 and IFIT2 were proposed to interact with stimulator of IFN genes (STING) (48), which recruits TBK1 (described above to bind IFIT3 by Liu et al.) and propagates phosphorylation of the transcription factor IRF3 (IFN regulatory transcription factor 3), activation of NF-κB, and IFN production together with MAVS (49, 50). However, in this case, over-expression of IFIT1 in HEK cells resulted in a decrease of IRF3 activation and IFNβpromoter activation in response to Sendai virus. Here, IFIT1 was described to disrupt the interaction of STING with MAVS or TBK1 (48). Given the conflicting results in pathway propagation and downstream immunological effects, more investigative work must be done on the individual IFITs, both *in vitro* and *in vivo*, in order to draw conclusions. Moreover, the extensive characterization of protein complexes formed by IFIT family members in various cell lines using affinity purification and mass spectrometry has failed to confirm any of these interactions.

## Conclusion

The IFIT family of proteins has recently been described as major players in anti-viral innate immunity, and their huge cellular abundance within the cell after ISG induction underscores their importance. Currently, the molecular mechanism for which high-resolution structure evidence exists, clearly defines a mechanistic role of IFITs by binding to foreign nucleic acid: interfering with viral processes, which expose a foreign 5′ configurations of RNA, such as protein translation. However, more work must be focused on determining the fate of the bound RNA: Are IFITs trafficking foreign nucleic acid for destruction, or is the natural turnover of IFIT naturally ridding the cell of foreign material? Moreover, greater detailed investigation of IFIT effect on host-translational machinery could lead to understanding of IFIT function in absence of virus infection. Following-up on these unanswered questions will better allow us to harness the potential activity of IFITs for anti-viral treatments by exploiting the direct effect of halting transcription of foreign nucleic acid, independently of sequence.

## Conflict of Interest Statement

The authors declare that the research was conducted in the absence of any commercial or financial relationships that could be construed as a potential conflict of interest.
